# Obliteration of Deformity from Depressed Cicatrices

**Published:** 1877-02

**Authors:** 


*Obliteration of Deformity from Depressed Cicatrices.—The British Medical Journal*—in alluding to a deformity which is often the result of neglected alveolar abscess, speaks of Mr. William Adams, having performed the following operations with great success. So many faces are rendered unsightly by these deep cicatrices, that any operation which results in the removal of the deformities must be a blessing to those afflicted by them. The operation is original, and consists in subcutaneously dividing all the deep adhesions of the cicatrix by a tenotomy-knife introduced a little beyond the margin of the cicatrix. and carried down to its base, so as to carefully and thoroughly evert the cicatrix which remains prominently raised.

Two hare-lip pins or fine needles are then passed through the base, at right angles to each other, so as to maintain the cicatrix in its everted and raised position, where it is so retained for three days. At the end of this time the needles are removed, and the somewhat swollen and infiltrated cicatricial tissue is allowed to settle gradually down to the proper level of the skin. He gives the full history of three such operations in his paper. The permanency of the cure is illustrated by cuts of two cases, in one of which the operation was done nine, and the other three years ago. The depressions seem to be completely obliterated.

---

